# 2D-DIGE as a strategy to identify serum biomarkers in Mexican patients with Type-2 diabetes with different body mass index

**DOI:** 10.1038/srep46536

**Published:** 2017-04-20

**Authors:** Erik E. Gómez-Cardona, Eric E. Hernández-Domínguez, Aída J. Velarde-Salcedo, Alberto-Barrera- Pacheco, Agustín Diaz-Gois, Antonio De León-Rodríguez, Ana P. Barba de la Rosa

**Affiliations:** 1 IPICyT, Instituto Potosino de Investigación Científica y Tecnológica A.C. Camino a la Presa San Jose No. 2055, Lomas 4a sección, San Luis Potosí, San Luis Potosí, 78216, Mexico; 2 Juridiscción Sanitaria No. 1, Centros de Salud San Luis Potosi, San Luis Potosi, Mexico; 3 Department of Genetics, University of Cambridge, Downing Street, Cambridge CB2 3EH, UK

## Abstract

Obesity and type 2 diabetes(T2D) are the most prevalent and serious metabolic diseases affecting people worldwide. However racial and ethnic disparities seems to be a risk factor for their development. Mexico has been named as one of the largest populations with the highest prevalence of diabetes and obesity. The aim of this study was to identify novel T2D-associated proteins in Mexican patients. Blood samples were collected from 62 Mexican patients with T2D and they were grouped according to their body mass index(BMI). A panel of 10 diabetes and obesity serum markers was determined using MAGPIX. A comparative proteomics study was performed using two-dimensional difference in-gel electrophoresis(2D-DIGE) followed by mass spectrometry(LC-MS/MS). We detected 113 spots differentially accumulated, in which 64 unique proteins were identified, proteins that were involved in metabolism pathways, molecular transport, and cellular signalling. Four proteins(14-3-3, ApoH, ZAG, and OTO3) showing diabetes-related variation and also changes in relation to obesity were selected for further validation by western blotting. Our results reveal new diabetes related proteins present in the Mexican population. These could provide additional insight into the understanding of diabetes development in Mexican population and may also be useful candidate biomarkers.

Obesity and diabetes have reached epidemic proportions in many countries worldwide showing a relationship with cardiovascular disease and affecting the quality of life and life expectancy^1^,^2^,^3^. Obesity is defined as excessive energy storage in white adipose tissue. Anyone who is overweight has a certain degree of insulin resistance leading to elevated fatty acids in the plasma, reducing glucose transport into the muscle cells, as well as increasing fat breakdown, and subsequently increasing hepatic glucose production^3^,^4^. Type 2 diabetes(T2D) is a metabolic disorder characterized by high blood glucose levels and defective carbohydrate utilization due to a relative or absolute deficiency of insulin or insulin resistance with impairment of β-cell function^5^. However, obesity-induced perturbations in metabolism have not been clearly established.

The World Health Organization(WHO) has estimated that 347 million people worldwide have diabetes and 3.4 million people died from consequences of high fasting blood sugar^6^. From those deaths, 80% occurred in low- and middle-income countries^7^. There are significant racial and ethnic disparities in the prevalence and trends of obesity and T2D, where non-Hispanic blacks and Mexican Americans seem to be at a higher risk than their non-Hispanic white counterparts^8^. In Mexico there are currently 10.6 million people who suffer from T2D and its complications^9^. The prevalence of overweight and obesity in Mexican adults is 71.3%(overweight 38.8% and obesity 32.4%) being more prevalent in women than in men^10^.

Although great advances have been made in the field of diabetes research, still clinical problems persist. Therefore, the identification of new biomarkers for early diagnosis and prediction would be useful to improved clinical outcome^11^. Theoretically, genetic alterations(DNA-based), differentially expressed transcripts(RNA-based), and differentially regulated proteins(protein-based) can all be used as biomarkers. Recent genome-wide association studies have reported many *loci* implicated in T2D pathophysiology. However, establishing a clear and direct causal relationship between common genetic variations and disease development is not trivial^12^. RNA levels do not necessarily correlate with protein concentration and proteins levels are difficult to predict from genomic patterns^13^.

Efforts have been made to identify and characterize the specific genes that contribute to T2D^14^, however those genes could not be necessarily expressed or might only be expressed as a result of complex interactions among socioeconomic influence, lifestyle factors, and genetic predisposition. Given that race and ethnic disparities are often viewed within a country-specific context^15^ that may put Mexican descendants at a greater risk of metabolic disease due to the epidemiological transition that changed the disease profile to a country dominated by nutritional related diseases such as obesity and diabetes^15^,^16^. The protein patterns in an organism are highly dynamic in relation to health and disease conditions and are tightly regulated by intra- and extra-cellular stimuli without any change at the genetic level^17^. Therefore, proteins offer high potential to serve as biomarkers for clinical applications^18^.

Proteomic analysis of blood has the potential to identify such biomarkers^19^, and the use of accessible human fluids such as plasma, which may contain tens of thousands of different proteins^20^. Serum and plasma have become the most widely used sample for diabetic biomarker studies^21^,^22^,^23^. Depletion of serum prior to proteomic analysis by immunoaffinity subtraction of specific proteins through the use of targeted polyclonal antibody columns and spin filters has recently emerged as a promising tool for serum prefractionation^19^. Serum depletion has become a mainstay of clinical proteomic studies, in particular in the area of biomarker discovery. As an analytical technique, two-dimensional difference gel electrophoresis(2D-DIGE) provides an insight into intact and fragmented protein species and also provides information regarding post-translational modifications. 2D-DIGE incorporates the use of fluorescent molecules(CyDyes), or fluors, that are used to pre-label samples prior to separation by 2-DE^19^, being a very sensitive gel-based proteomic technique that is unique through the utilization of fluorescently labelled samples on the same gel, and the application of an internal standard for intra- and inter-gel comparisons and normalization^24^. This technique has been used for studies of hepatocellular carcinoma^25^, alterations in human endometriosis^26^ and patients with diabetes and cardiovascular disease^27^.

We are not aware of previous studies to analyse the proteomic changes in Mexicans patients with diabetes in relation to their obesity state. The aim of the present study was to use a quantitative proteomic approach employing 2D-DIGE and LC/MS-MS to compare serum proteome profiles from Mexican patients with four well-defined obesity degrees: normal weight, overweight, obesity I and obesity II/III. We report for the first time potential protein biomarkers that could play a key role in the development of diabetes in Mexicans. Serum biomarkers with the ability to differentiate between these phenotypes would have clinical relevance with regard not only to diagnosis, but also to long-term prognosis.

## Results

### Patients Serum Biomarkers

The patients with diabetes were grouped according to their body mass index into four groups: normal weight(NW), overweight(OW), obesity I(ObI), and obesity II and III(ObII/III). A control group(Ctrl) of healthy volunteers was also included. Additional characteristics of these groups are presented in Supplementary Information Table S1. A panel of 10 serum pre-defined diabetes biomarkers was measured using MAGPIX technology. The generated data give us more valuable information about the group’s health conditions.

Leptin and ghrelin are hormones that have been recognized to have a major influence on energy balance. Table 1 shows that leptin increased as BMI increased from 1717.8 pg/mL(NW) to 6482.1 pg/mL(ObII/III), while a significant reduction in ghrelin levels was found in the groups ObI(329.8 pg/mL) and ObII/III(287.1 pg/mL) compared to the Ctrl group(360.2 pg/mL).

Other molecules produced by adipose tissue(adipokines) such as visfatin and resistin, also related to diabetes and obesity were measured. Visfatin concentrations compared to the Ctrl group(882.5 pg/mL) increased in relation to obesity degree(875 to 1798.8 pg/mL). Differences in resistin levels were detected but there was no clear relationship between their concentration and obesity degree, lower levels than the Ctrl group were found in ObI group(1162.9 pg/mL) and the highest levels were detected in ObII/III group(1722.8 pg/mL). The plasminogen activator inhibitor 1(PAI-1), an important inhibitor of the fibrinolytic system, which is also an adipokine, showed an increased level in relation to the obesity degree from 8591.6 pg/mL in Ctrl group to 12323.1 pg/mL in ObII/III.

Hormones involved in glucose regulation in blood were also measured; glucagon is the molecule that raises glucose concentration in the bloodstream and insulin is involved in glucose uptake in peripheral tissues. Statistically significant changes in glucagon(at *p* ≤ 0.01) were only detected in OW and ObII/III groups(Table 1), while insulin significantly increased from 59.9 pg/mL(NW group) to 212.3 pg/mL in ObII/III group. The C-peptide generated by cleavage during proinsulin processing was also increased as insulin increased, showing values of 8472.2 pg/mL in NW to 16711.3 pg/mL in ObII/III(Table 1).

Additionally, incretins(molecules secreted in a nutrient-dependent manner) were measured due to their anti-diabetic properties and the relation they have with insulin and glucagon secretion. A reduction in GIP(glucose dependent insulinotropic polypeptide) levels was detected in 8NW and ObI groups(45.88 pg/mL and 43.1 pg/mL, respectively) compared to the Ctrl(61.18 pg/mL) while an increment was observed in ObII/III group(94.0 pg/mL). The Glucagon-like peptide 1(GLP-1) was increased only in OW group(139.5 pg/mL) compared with the Ctrl group(93.0 pg/mL). Results did not show a correlation between incretin levels and BMI. In addition serum dipeptidyl peptidase-IV DPPIV activity was measured(Table 1) in order to analyse if the enzyme in charge of incretin inactivation was related to this behaviour. Reduced DPPIV activity was detected in NW and OW groups. This might be related to the changes observed for GLP1 but not for GIP.

### Serum Depletion and Identification of Differentially Accumulated protein

To reduce the complexity and enrich the proteins that might be used as biomarkers, immunoaffinity depletion using a MarsHu14 column was carried out, and two fractions were collected. One included ~94% of the proteins in serum or high-abundance proteins(HAP) and the other included the low-abundance proteins(LAP). As shown in Supplementary Information Fig. S1, the removal of HAP species allowed the significant enrichment of LAP species, resulting in clearer 2-DE maps and increasing the number of detectable spots.

**Figure 1:**
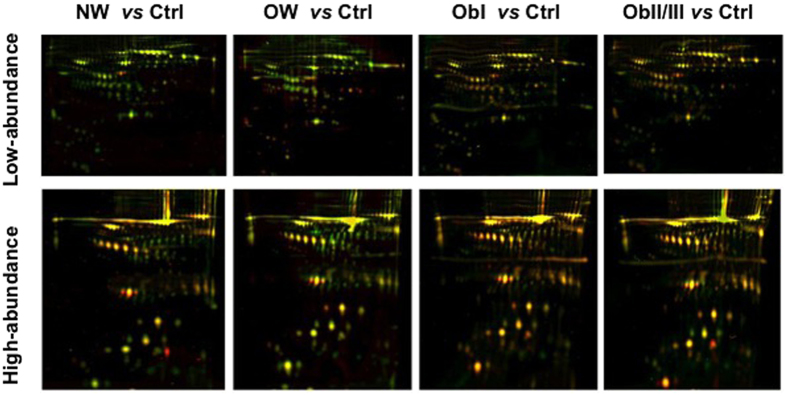
Representative merged 2D-DIGE gels images from low- and high-abundance serum proteins. Spots that are only present in the control group are shown in green, unique spots in groups with diabetes are shown in red and spots present in both conditions are shown in yellow. Ctrl = control, NW = normal weight, OW = overweight, ObI = obesity I, and ObII/III = obesity II and III.

**Figure 2:**
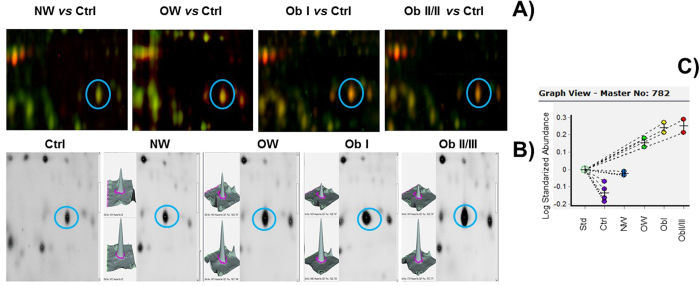
Differentially accumulated spots in low-abundance proteins identified by Biological Variation Analysis. (**A**) Merged image of the spot of interest showing differential accumulation. Full-length image is shown in Fig. 1.(**B**) Image view and 3D view showing relative abundance of a spot between the control group(superior panel) and test groups(lower panel).(**C**) Graph view in DeCyder v7.2 presenting the log standard abundance of the spot through the groups: Std = standard, Ctrl = control, NW = normal weight, OW = overweight, ObI = obesity I, and ObII/III = obesity II and III.

Once obtained HAP and LAP fractions from each of the groups, proteins were labelled with CyDyes, for 2D-DIGE. As a strategy to remove gel-to-gel variation gel-to-gel variation, one problem sample, one control sample, and one internal standard were run in a single gel. The order in which proteins were labelled is shown in Supplementary Information Table S2. A total of 1062 protein spots were detected in 2D-DIGE gels for the LAP fraction and 600 in the HAP fraction. Representative 2D-DIGE gels of each condition are shown in Fig. 1. To confirm correct matching of spots across the gel images an overlapping of the internal standard image to the master gel was obtained using the match vectors to lead warping of the images and making manual corrections when needed until spot matching was acceptable. A differential spot was defined when significant changes in the logarithm of the normalized-protein abundance were observed(One-way ANOVA at *p* < 0.05). In Fig. 2A is presented an example of a differentially accumulated spot with a clear difference between the control group and the groups with diabetes. To measure the abundance changes between spots a Biological Variation Analysis(BVA) was done using the DeCyder software, which allows spot detection, background subtraction and in-gel normalization(Fig. 2B and C). In total, 113 spots(56 in HAP and 57 in LAP) were determined to have statistically significant differential accumulation. These spots were excised from a preparative Coomassie Brilliant Blue-stained gel and subjected to in-gel digestion and peptides purification for ESI-MS/MS analysis. After *m*/*z* data analysis was carried out, it was found that several spots contained more than one protein; overall 64 unique proteins were identified in both fractions(Supplementary Files S1 and S2).

Following MS analysis, all successful protein identifications were subjected to functional classification using GO annotations. Identified proteins were classified in order to gain more information about the molecular function and biological processes in which they are involved(Supplementary Information Fig. S2). The identified proteins were mainly classified within catalytic activity, enzymatic regulation and binding activity, and fulfil their function in metabolic processes, biological regulation, stimuli responses and immune system processes.

### Pattern of variation and grouping differentially accumulated proteins

**Figure 3:**
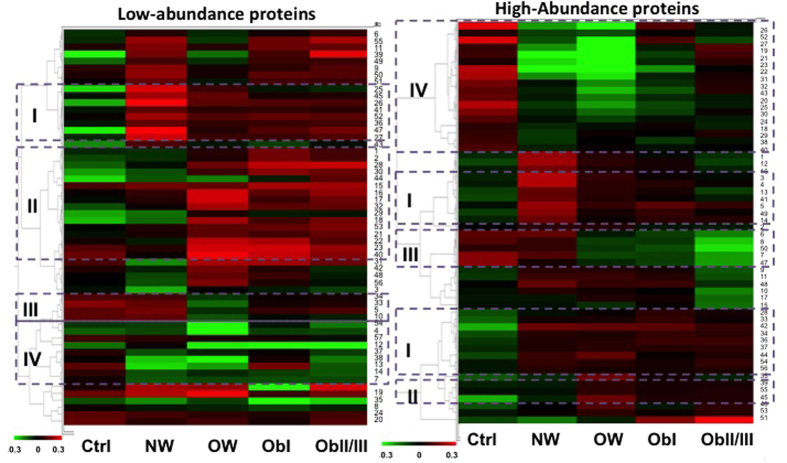
Heat map representation of the differentially accumulated protein spots from low- and high-abundance protein fractions. Each column shows a different group of the study and the rows show single spots. The increase and decrease in the abundance of one spot is indicated based on a relative scale(−0.3 to 0.3), shown from red to green. Dashed boxes show groups of spots with similar changes in abundance. I to IV clustered groups; Ctrl = control, NW = normal weight, OW = overweight, ObI = obesity I, and ObII/III = obesity II and III.

To evaluate similar patterns of regulation and meet the trend of differential proteins and their behaviour in different degrees of obesity, an extended data analysis(EDA) was carried out. This analysis involves the application of algorithms that can help to find data subsets(clusters) in the patterns of differentially accumulated proteins for each condition. The results of the BVA analysis were processed to generate the heat maps at the same time as the hierarchical grouping with similar behaviour(Fig. 3). Pearson correlation coefficient was used for distance metrics and average linkage method was employed to define distance among clusters. Data subsets that include spots with similar trends through the patients are shown in dotted boxes. Subsets I and IV include spots with increased and decreased abundance, respectively; in diabetic patients while groups II and III have proteins that might not be related to the diabetic state because they show abundance changes only in OW and obesity, but not when compared to NW group. It is important to mention that in the HAP, the subset I was divided which might indicate that there are two different patterns among the spots that increase abundance in diabetic patients, it is possible that other factors associated with diabetes affect in different rates the proteins accumulation in this subset creating data separation. In order to obtain a better definition in the group’s classification, a partition clustering analysis was carried out and self-organizing maps(SOM) were defined. Several partition-clustering analyses were done by changing the number of groups expected, the number of iterations per analysis, and the distance metrics. Pearson correlation coefficient was the measurement used for association among the spots. It is possible that some clusters contain zero entities, which indicates that the real number of clusters might be smaller(Supplementary Files S3). Changing the number of expected clusters, in order to improve the quality parameters(q value), this could even accept clusters with zero entities. For each clustering event, the quality parameters such as cluster validity score and the quality measurements were compared. The information here reported is the result of the analysis with the best quality values. As a result, 12 clusters for LAP and 9 clusters for HAP were obtained with the higher grouping quality value(q) possible. All the members included in the same cluster had similar tendencies; contiguous groups had similar patterns while distant clusters had different patterns(Supplementary File S3). The possible tendencies observed in the clusters are: a) proteins related to obesity but not to diabetes; b) proteins related to diabetes but not to obesity, c) proteins related to both, diabetes and obesity conditions, and d) proteins with punctual variation(those with no particular tendency), some examples of these tendencies are shown in Supplementary Information Fig. S3.

### Validation of Differentially accumulated serum Proteins by Western blot analysis

**Figure 4:**
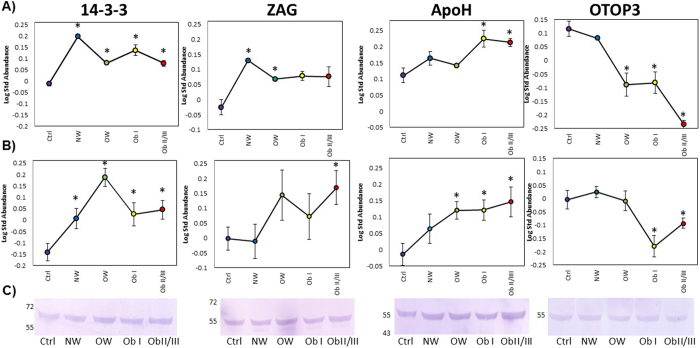
Validation of candidate biomarkers by western blotting. (**A**) Image view of the differentially accumulated spot where the protein was detected.(**B**) Densitometry analysis of WB. The graph shows the log-standardized abundance of the bands detected by WB against 14-3-3, ZAG, ApoH and OTOP3 proteins through the groups.(**C**) Serum levels of the proteins of interest examined by WB in the pooled serum samples for each group. The error bars indicate standard error of measurements performed at least in triplicate. Asterisks show significant differences at *p* < 0.05. Full-length blots are presented in Supplementary Fig. S5.

Proteins in the clusters were compared with the lists of diabetes-related proteins generated by the Human Diabetes Proteome Project(HDPP). A total of 27 identities were not previously included in such lists(Table 2). Those with a clearer trend of increase or decrease in diabetes and/or obesity were considered as proteins of interest that might act as candidate biomarker. Proteins of interest were identified in both fractions, HAP and LAP, we focused on 4 proteins(Fig. 4A), two of them located in a cluster that included diabetes related proteins: 14-3-3 protein beta/alpha(spots 22 and 24 from HAP) and Zinc-alpha-2-glycoprotein(ZAG; spots, 26, 36, and 37 from LAP); one protein included in clusters with tendencies related to diabetes and obesity, Beta-2-glycoprotein or ApoH(spots 40, 47, and 51 from LAP), and Otopetrin 3(OTOP3, spot 37 from HAP) that showed an obesity-related behaviour. The accumulation pattern of these four proteins was confirmed by western blotting analysis(Fig. 4B and C). Results observed for 14-3-3, ApoH and OTOP3 confirm the behaviour observed in 2D-DIGE BVA analysis and correlate also with the tendency observed in the partitioned clustering analysis. Accumulation of 14-3-3 was significantly increased in diabetic patients when compared to the Ctrl group, ZAG showed an increase in OW and obesity groups as observed in DIGE but the increase was significant only in the ObII/III group. ApoH accumulation was significantly increased in relation to BMI, showing statistical differences in OW and obesity groups. OTO3 showed a significant decrease in ObI and ObII/III groups. Validation for individual samples was carried out for 14-3-3 protein(Supplementary Information Fig. S4).

## Discussion

Leptin acts as part of a signalling pathway that regulate the size of the body fat depot, acting directly or indirectly on the central nervous system to inhibit food intake and/or regulates energy expenditure^28^. By contrary, ghrelin concentrations are negatively correlated with BMI in humans and insulin resistance^29^, the observed increased levels of leptin and decrease levels of ghrelin might be associated to the fat mass depots in obese groups(Table 1). Reports have indicated that insulin could induce leptin secretion and production event though body fat is the determinant factor of circulating leptin^30^. It has been also reported that ghrelin levels also increased when obese humans lose weight and decrease when they gain weight^31^. However, several human studies have shown conflicting results. Not only the size and frequency of meals have an effect on circulating leptin and ghrelin levels, but also the composition of a meal is a determinant of leptin and ghrelin levels in humans^31^,^32^. Our results(Table 1) might be affected by these situations, despite the circulating levels of the hormone leptin were increased in all obese groups, the hormone ghrelin was decreased in obese subjects(ObI and ObII/III groups), but not in OW group.

Visfatin and PAI-1 are adipokines that play important roles in inflammatory processes and both, have been considered as cardiovascular relevant molecules response. Visfatin is considered as a proinflammatory cytokine implicated in the pathogenesis of a cluster of disorders including hyperlipidemia, hypertension, and increased risk of cardiovascular diseases. Additionally elevated levels of this molecule have been reported in Type-1 diabetes, T2D and gestational diabetes brown^33^,^34^. PAI-1 is an important inhibitor of the fibrinolysis system, so high levels of this molecule may suppress the degradation process of plasminogen to form plasmin by inhibition of urikinase, representing a significant risk factor for macro-vascular complications and cardiovascular disease, particularly in patients with diabetes. In patients with impaired glucose tolerance, elevated concentrations of PAI-1 were observed through the development of diabetes^35^. In this study the levels of visfatin and PAI-1 were higher in overweight, ObI and ObII/III groups(Table 1), in agreement with previous reports, suggesting that these proteins could be a promising biomarker in cardiovascular disease related to metabolism alterations.

The connecting peptide or Pep-C is a short 31 amino acid protein that connects insulin A-chain to the B-chain in the proinsulin molecule. It is now being considered as a bioactive peptide with endocrine functions due to its ability to interact by binding to the surface of neurons, fibroblasts and renal tubular cells^36^. In this work, the levels of C-peptide as well insulin increased in patients with OW and obesity, indicating an elevated production of insulin that might be related to the insulin resistance generally observed in diabetic patients.

Incretin hormones, GIP and GLP-1, are secreted in response to food intake and act on pancreatic β-cell receptors, stimulating insulin release in a glucose dependent way^37^. Incretins degradation is carried out by the enzyme dipeptidyl peptidase-4(DPPIV), which is nowadays used as therapeutic target as an alternative to control glucose levels. Some reports indicate that DPPIV has increased activity in patients with diabetes, causing low incretins levels^38^. However, only NW and ObI groups showed a reduction in GIP levels. On the other hand, Firneiszy *et al*.^39^ reported that patients with T2D showed lower levels of DPPIV activity with respect to the healthy volunteers. This is in agreement with our results, which showed a reduction in DPPIV activity in patients with diabetes but incretins levels were higher only in OW group for GLP1 and ObI and ObII/III groups for GIP. There was no clear correlation of incretin concentrations neither to the obesity rate or DPPIV activity, reinforcing the idea that DPPIV activity is not necessary a reflex of the incretins degradation grade *in vivo*^40^.

Biomarker discovery in serum using proteomic approaches involves some technical challenges due to the complexity of the sample. As the concentration range of the proteins fluctuates from pg/mL to mg/mL the signal of those at low concentrations will be masked by the abundant proteins in serum. To reduce the complexity and enrich the low-abundance proteins that might be used as biomarkers, immunoaffinity depletion using the MARSHu14 column was carried out.

Rifai *et al*.^41^ defined that the essential processes in biomarker involves long path from discovery to validation and finally the clinical application. In the first step, the Discovery phase, simple comparisons between disease and healthy conditions are recommended. At this step the variability should be reduced because the huge number of analytes and the complexity of the techniques may complicate the discovery of the biomarkers that is why the use of pools during this phase is frequently observed^42^,^43^,^44^. Some information could be lost, for example, the contribution of some variables such as gender, age, genetics, and the environment, but variability is reincorporated in “Verification” phase where the analysis is extended to a larger number of human samples, with representative patients for each variable not considered in the Discovery phase. Once a protein has successfully passed through all the steps until validation a clinical evaluation is needed. These advanced clinical trials involve patients across different health care and geographical settings and are oriented to define the diagnostic accuracy and predictability of the final biomarker. Studies in different populations are useful tools to learn the scope of the biomarker.

In this study, we show the result of the Discovery and Qualification steps in biomarker discovery and we report a total of 113 differentially accumulated protein spots in serum from Mexican patients with diabetes compared to the control group. Changes in the abundance of these proteins were analysed in relation to BMI and 64 unique proteins were successfully identified by MS/MS. The identified proteins were classified according to their biological process showing a similar distribution to those biomarkers previously reported for serum and other biofluids from diabetic patients^45^, except for metabolic processes, which are over-represented in the present study. When comparing the 64 proteins identified with the HDPP lists, it is important to mention that 37 proteins already reported in European population were detected in Mexican patients, which shows a validation of our study but also that those proteins do not have any relation with ethnicity. However, 27 proteins identified in this work were not previously reported and it is interesting that most of these proteins belong to the metabolic process, which may be related with the genetic background, being ethnicity important in the disease development. Four of these proteins(14-3-3, OTP3, ApoH, and ZAG) showing a clear tendency related to the disease were selected for further validation.

In mammals, the 14-3-3 proteins family are molecular adaptors that comprise seven isoforms(α/β, ε, η, γ, τ/θ, δ/ζ, and σ). They are crucial regulators of intracellular signalling pathways and each isoform has unique and fundamental roles^46^. Bioinformatics studies have predicted that 14-3-3 proteins have been associated with T2DM as well as Alzheimer-type pathology^47^. Ramm *et al*.^48^ indicated that insulin-dependent association of 14-3-3 with AS160(Akt substrate of 160 kDa) plays an important role in GLUT4 trafficking in adipocytes. According to our results 14-3-3 protein showed association to diabetes because this protein was found accumulated in 2D-DIGE and was corroborated by western blot in both the pools and in individual samples(Fig. 4 and Supplementary Information Fig. S4) and as far we know, this is the first time that 14-3-3 proteins are found *in vivo* in relation with diabetes.

Zinc-α2-glycoprotein(ZAG) has recently been reported in newly-diagnosed T2D^49^. ZAG has been proposed to play a role in the pathogenesis of insulin resistance indicating that ZAG may be an adipokine associated with insulin resistance and sitagliptin treatment^50^. Our 2D-DIGE results predicted a diabetes related tendency for ZAG but after validation ZAG was increased in patients with OW or obesity groups, indicating that this protein is associated to obesity state rather than diabetes.

Apoliprotein H(ApoH), also known as β2-glycoprotein I, is a 44-50 kDa single-chain glycoprotein plasma protein that also binds other negatively charged molecules such as DNA. ApoH plasma concentrations are strongly associated with metabolic syndrome alterations and vascular diseases in T2D patients and could be considered as a clinical marker of cardiovascular risk^51^. ApoH was a differentially protein that is accumulated in relation to the BMI(Fig. 4), which might be associated to the increased levels of the other cardiovascular risk molecules measured in this study, such as PAI-1 and visfatin^52^.

Contrary to the other validated molecules, otopetrin 3 showed decreased levels in an obesity-related behaviour in both, 2D-DIGE and western blot(Fig. 4). The *otop* gene family in most vertebrates is comprised of three members clustered on two chromosomes: *otop*1 and the paralogous tandem genes *otop*2 and *otop*3. Otop1 was identified as a component of a counter inflammatory pathway stimulated by tumour necrosis factor-α in cultured adipocytes^53^ and its expression was markedly increase in obese mouse white adipose tissue(WAT). Otop defines a unique target of cytokine signalling that attenuates obesity-induced adipose tissue inflammation and plays an adaptive role in maintaining metabolic homeostasis in obesity^53^. Our experimental design allowed the identification not only of diabetes related proteins, but the results also suggest that OTP3 could act as an obesity-related biomarker in humans as OTP1 does in mouse.

Validated proteins represent an example of the molecules involved in the complex network behind diabetes pathogenesis and its association to obesity. The identification of these proteins was facilitated by the application of 2D-DIGE techniques, which is more sensitive than conventional gel-based methodologies but mainly by the fact that the population under study has a unique genetic background derived from the admixture of European, Mesoamerican-Indigenous and African populations.

In conclusion, some of the proteins reported here have been previously related to diabetes but this is the first time they were identified in Mexican patients using proteomics tools. Our results, as part of the discovery phase in the biomarkers development, are the starting point for the next step, the validation phase. In validation work, additional analysis will be necessary in a larger population in order to discriminate those proteins that could be related to other factors such as age, gender and genetic background. Nonetheless, the proteins detected here and which are not included in HDPP lists are particularly important because they give new insights on the disease development and may act as protein biomarkers specifically for the Mexican population, which could be related to the ethnicity predisposition and might help to develop new therapies or better diagnostic tools.

## Materials and methods

### Ethic statement

The study was conducted according to the Mexican General Health Law and the Official Mexican Norm NOM-012-SSA3-2012, which establish the criteria for research project execution for human health. This norm is based on the Declaration of Helsinki and the Ethics Committee Research of Institute for Social Security and Services for State Workers approved the protocol(ISSSTE-SLP-Mexico, Reg. COFEPRIS 123301538X0054) with reference MX-M453N.

### Participants selection

Participant’s recruitment protocol included approaches based on searches of patient databases of preventive medicine area from the Clinic Pedro Bárcena Hiriart-Institute for Security and Social Services of State Workers(ISSSTE)-San Luis Potosi, S.L.P., Mexico. Participants, both male and females, with at least 5 years since being diagnosed, according to the recommendations and classification of T2D published by the International Diabetes Federation(http://www.idf.org), using hypoglycemic drugs but not insulin, and without any hepatic, kidney or cardiovascular disease were included in this study. Patients and control participants selections was carried out following the recommendations described elsewhere^54^. Patients were clearly identified by the medical team in charge as well as by the nutritionist and nurses who have followed their diabetic state. Once patients were identified in the database, they were invited to participate in the study. A total of 62 patients with T2D were included and all of them signed an informed written consent. Healthy volunteers(n = 23), with normoglycemic values and normal weight who agreed to participate in this study were also included(control group).

### Anthropometric and Physical Measurement

Physical measurements of height(H) and weight(W) were assessed using standardized methods, with participants dressed in light clothing and barefoot. Height(cm) was measured to the nearest 0.5 cm using a portable stadiometer against the wall, with participants standing in an upright position on a flat surface without shoes, with the back of the heels and the occiput on the stadiometer. W(kg) was measured to one decimal place using a Tanita electronic digital scale(Arlington Heights, Illinois, USA). Body mass index(BMI) was calculated using the averages of the H and W as follows: [weight(kg)].[height(m^−2^)]. Patients were grouped according to the BMI into: normal weight(BMI from 18.5 to 24.9 kg/m^2^), overweight(BMI ≥ 25.0 kg/m^2^), obesity I(BMI 30.o to 34.9 kg/m^2^), obesity II and III(BMI 35.0 to ≥ 40.0 kg/m^2^) and control(Ctrl) group, all were classified all as normal weight persons.

### Blood sample collection

Blood samples from all participants, the control and patients groups were taken in the morning in fasting conditions in August 2013. Blood samples were collected into vacutainer tubes(Becton, Dickinson & Company, New Jersey, USA) without anticoagulant and serum was separated by centrifugation at 300 × g for 20 min. Serum pool was generated for each group and pools were used in all following analysis. All samples were stored at −80 °C until analysis.

### Serum Biomarkers Levels and DPPIV activity

In order to measure serum concentrations of ten diabetes-related peptides and evaluate if the study groups presented the typical behaviour related to their condition. Glucagon, Insulin, C-peptide, the incretins Glucagon-like peptide-1(GLP-1) and Gastric inhibitory polypeptide(GIP), and the adipokines Resistin, Visfatin, Leptin and plasminogen activator inhibitor-1(PAI-1) were quantified using an optimized multiplex test BIO-PLEX Pro Diabetes Assay(Bio-Rad, Hercules, CA, USA) in a multiple reader Bio-Plex MAGPIX(Bio-Rad). All samples were analysed in triplicate.

The activity of the enzyme responsible for inactivation of incretins was analysed in triplicate for each of the groups in the study using a continuous monitoring assay in a microplate reader at 405 nm for 30 min at 37 °C. On each well 5 μL of serum were mixed with 45 μL of the assay buffer(10 mM Tris-HCl, pH 7.6) and 50 μL of the substrate 1 mM Gly-Pro-p-nitroanilide tosylate(Gly-Pro-PNA, Sigma-Aldrich, St. Louis, MO, USA). The activity of the DPPIV was calculated with the values at 30 min and presented as U/L(nmol/mL/mL) of hydrolyzed Gly-Pro-PNA.

### Affinity Depletion of Serum Samples

High-abundance proteins in serum were depleted using a multiple affinity column MARS Hu-14 4.6 mm × 100 mm(Agilent Technologies, San Diego, CA, USA) which removes in a single step the 14 most abundant proteins in serum(albumin, IgG, antitrypsin, IgA, transferrin, haptoglobin, fibrinogen, alpha-2 macroglobulin, alpha-1-acid glycoprotein, IgM, apolipoprotein AI and AIII, complement factor C3 and transtiretina). Samples were processed according to the manufacturer’s instructions using pooled samples of the groups. Two fractions were collected, the first flow-through fraction with the low-abundance proteins in serum(LAP) and the second eluted fraction with the high-abundance proteins(HAP). Both fractions were passed through a 5 kDa molecular weight cut-off(MWCO) concentrator at 7500 × *g* until the volume was reduced and the buffer was then exchanged to 10 mM Tris-HCl(pH 7.4) supplemented with complete protease inhibitor cocktail(Roche Molecular Biochemicals, Mannheim, Germany). The concentration of proteins in the fraction was measured in a microplate reader using the Bradford method(Protein Assay, Bio-Rad) with BSA to create a standard curve.

### Reconstitution of CyDyes and Labeling of Protein

Each CyDye(DIGE Flours, GE, Healthcare, Uppsala, Sweden), was resuspended in 5 μL of anhydrous N,N-Dimethylformamide(DMF, 99.8%, Sigma-Aldrich, St. Louis, MO, USA) to give a final dye concentration of 1 mM. A working solution of 400 pmol/mL of each CyDye was generated by dilution of the stock with dimethylformamide. This solution was used to label the protein samples. Depleted serum protein(50 μg) samples were labelled with 400 pmol of dye for minimal labelling. The labelling reaction was conducted at 4 °C in the dark for 35 min. The reaction was quenched by the addition of 1 μL of 10 mM lysine(Sigma) for 10 min at 4 °C in the dark. A pooled internal standard, which contained equal parts of the samples to be compared, was used in order to eliminate gel-to-gel variation and facilitate comparison. Three different samples were included on the same 2-DE gel, two of them for the group samples and one the Std. A dye-swap was performed to control for preferential labelling; the combination of the labelled proteins from the different groups and Std for each gel is shown in Supplementary Information Table S2. The samples were then pooled together and the total volume of the samples as made up to 450 µl with rehydration buffer(8 M urea, 0.5% CHAPS, 0.2% DTT, 0.2% Pharmalyte pH 3–10).

### 2D-Difference Gel Electrophoresis(2D-DIGE)

Labelled samples were used for in-gel rehydration overnight at room temperature using 24 cm Immobiline DryStrips with pH 4–7(ReadyStrip IPG Strip, Bio-Rad) for the LAP samples. The HAP fractions were processed in 13 cm linear gradient strips in the same pH range(GE Healthcare). Rehydrated strips were focused using an Ettan IPGphor(GE Healthcare). Following isoelectric focusing, each strip was equilibrated with a reducing equilibration buffer(6 M urea, 50 mM Tris-HCl pH 8.8, 30%(v/v) glycerol, 2%(w/v) SDS, 1%(w/v) DTT) for 15 min followed by equilibration with and alkylation buffer(6 M urea, 50 mM Tris-HCl, pH 8.8, 30%(v/v) glycerol, 2%(w/v) SDS, 4.8%(w/v) IAA) for 15 min. The strips were then placed on top of 12% SDS-PAGE gels and sealed with an agarose sealing solution(25 mM Tris, 192 mM glycine, 0.1% SDS, 0.5%(w/v) agarose, 0.02% Bromophenol blue). Separation of the protein in the second dimension was carried out in an Ettan Dalt-six electrophoresis tank(GE-Healthcare) in a 12% SDS-polyacrylamide gels. Resulting gels were scanned using a Pharos FX System(Bio-Rad) at 100 μm resolution.

### Mass Spectrometry Analysis

Spots that were found to be statistically significant were manually excised from a preparative gel of pooled samples from both, low- and high-abundance fractions. The preparative gels were obtained by conventional 2-DE, using identical conditions as before, with 1 mg of pooled depleted samples for each fraction and stained using Coomassie Blue. Each spot was distained, reduced(10 mM dithiothreitol in 25 mM ammonium bicarbonate), alkylated(55 mM iodoacteamide), and digested overnight at 37 °C with trypsin(Promega, Madison, WI, USA). Tryptic peptides were separated using the nano-ultra performance liquid chromatography(nano-Acquitiy UPLC, Waters, Milford, MA, USA) Tryptic peptides were separated by nanoscale liquid chromatography(nanoAquity UPLC system, Waters, Milford, MA, USA). Each sample was loaded onto a Symmetry C_18_ precolumn(5 μm, 20 mm × 180 μm, Waters) and BEH130 C_18_ analytical column(1.7 μm, 100 mm × 100 μm, Waters), which were connected directly to the ESI source of the SYNAPT-HDMS Q-TOF(Waters). The spectrometer was operated in V-mode in the positive mode and calibrated with [Glul]-Fibrinopeptide B from *m*/*z* 50 to 2422. MS and MS/MS spectra were acquired alternating between low-energy and elevated-energy mode acquisition(MS^e^). Mass spectrometry results were processed to generate PKL files. Proteins were identified using MASCOT search engine v2.5(Matrix Science, London, UK). Searches were conducted against the *Homo sapiens*(human) database from SwissProt(20200 sequences, January 2015). Trypsin was used as the specific protease and one missed cleavage was allowed. The mass tolerance for precursor and fragment ions was set to 10 ppm and 0.0.5 Da, respectively. The protein identification reporting criteria included at least one MS/MS spectrum matched at 95% level of confidence.

### Statistical and Bioinformatics Analysis

GradPad Prism v6.0 was used for Bioplex MAGPIX quantitation and WB analysis to define significant differences between groups with diabetes *vs* control group were determined using a one-way ANOVA comparison and in order to identify differences between a specific group and the control group, the Student’s t-test was applied. Results were considered significant if *p* < 0.05. Quantitation of diabetes-related peptides was done in triplicate and results were expressed as the mean value ± standard deviation.

Gel images were processed in the DeCyder 2D Differential Analysis software v7.2, which uses a number of complex algorithms designed specifically for multiplexed 2D images experiments. The image analysis involves the following processes: spot detection, background subtraction, in-gel normalization, gel artefact removal, gel-to-gel matching and statistical analysis. Numeric values form the image are obtained by analysing the pixels in the image, each pixel has an intensity value that describes how bright the pixel is, which represents a measurable physical property. The intensity value in the following analysis the average for the whole area covered by the pixels that constitute one defined spot. An experimental design that uses the same internal standard on every single gel of the study was used. Normalization for the spot intensities was done by comparing individual intensities against the standard value and generating a fold change referred to the standard.

A Biological Variation Analysis was acquired for the normalized volume of the spots. Multiple images from different gels were matched to provide the protein expression level between multiple groups and identify differentially accumulated protein spots with the help of one-way ANOVA and the multiple comparison tests included in the statistical package. As observed for other biomarker discovery approaches in serum using 2D-DIGE and depletion systems^55^,^56^, protein abundance changes were considered significant if *p* < 0.05. False Discovery rate(included in the software) was applied as multiple testing correction method to adjust values for each spot to keep overall rate error as low as possible. The cut-off value was 0.02 while using this adaptive procedure^57^.

The differential spots were processed in the Extended Data Analysis module included in the DeCyder software with the purpose of generating clusters with similar tendency through the groups using the pattern and partition analysis also included in the software.

The identified proteins were additionally classified into different categories according to their molecular function and the biological processes in which they are involved, this was accomplished thanks to the information contained in the Gene Ontology database(http://www.geneontology.org/).

### Western Blotting

To validate the 2D-DIGE results, target proteins underwent Western blotting as reported before Huang *et al*.^25^ Briefly, after diluting crude sera 10-fold with Milli-Q water(without depleting abundant proteins) quantitation was carried out and 30 μg of serum were mixed with 5X loading buffer(2 μl) and loaded in each lane of one 12% SDS-PAGE with 4% stacking gel. SDS-PAGE was run in triplicates to provide a gel for Coomassie Blue staining for determining the amount of protein loaded for each sample. As no reliable internal control protein was available for Western blot analysis of serum samples, a loading control sample was generated by pooling all of the samples from all groups in equal volumes for each gel. Electrophoresis was carried out in a semi-dry chamber(Bio-Rad). The 1-DE gels were run at 15 V for 45 min, and then proteins were transferred to polyvinylidene fluoride membranes(PDVF, Bio-Rad) in ice-cold transfer buffer(25 mM Tris, 192 mM glycine, 20%(v/v) methanol). The separated proteins were transferred to polyvinylidene fluoride membranes(PVDF, Bio-Rad). Membranes were blocked in PBST(170 mM NaCl, 50 mM Tris, pH 7.4, 0.1% Tween) containing 5% non-fat milk overnight at room temperature. The membranes were then washed in PBST(3 × 10 min washes) and probed with appropriate antibodies for 2 h at room temperature. Antibodies for the target proteins were: 14-3-3 at 1:50000 dilution, ApoH and Zag 1(Santa Cruz Policlonal IgG anti-rabbit) at 1:1000, Otopetrine 3 and Zag 1(AbCam polyclonal IgG rabbit) at 1:1000 dilution, incubation for 1 h after three washes with PBST. The secondary antibody was a 1:10000 dilution of anti-rabbit IgG coupled to alkaline phosphatase(Sigma), incubated for 1.5 h, followed by 3 washes before proteins bands were revealed with NBT/BCIP solution. The membranes were scanned and the density of each band was measured with Quantity One(BioRad) for semi-quantitative analysis. The loading control sample in each gel was used as the standard for normalization. Individual sample validation was done for 14-3-3 protein. Eleven blood samples of each group(Ctrl, NW, OW, ObI and ObII/III) were randomly selected for western blot analysis. After densitometry analysis, the accumulation level of 14-3-3 was obtained as the average value for the patients normalized with the total protein amount per sample.

## Additional Information

**How to cite this article:** Gómez-Cardona, E. E. *et al*. 2D-DIGE as a strategy to identify serum biomarkers in Mexican patients with Type-2 diabetes with different body mass index. *Sci. Rep.* **7**, 46536; doi: 10.1038/srep46536(2017).

**Publisher's note:** Springer Nature remains neutral with regard to jurisdictional claims in published maps and institutional affiliations.

## Supplementary Material

Supplementary Information

Supplementary Files

## Figures and Tables

**Table 1:** Diabetes marker concentrations and DPPIV activity in serum of Mexican patients with diabetes according to their BMI^a^.

Serum Marker	Control Group	Patients with diabetes			
		NW	OW	ObI	ObII/III
Leptin	2505.4 ± 72.4	1717.8 ± 37.9**	2698.2 ± 65.9*	3987.6 ± 108.2**	6482.1 ± 223.0**
Ghrelin	360.2 ± 7.1	393.2 ± 11.8**	552.6 ± 12.6**	329.8 ± 11.9*	287.1 ± 4.6**
Visfatin	882.5 ± 37.3	875 ± 12.6	2190.2 ± 54.2**	950.3 ± 25.1*	1798.8 ± 62.4**
Resistin	1471.5 ± 45.2	1384.2 ± 40.2	1664.5 ± 69.7**	1162.9 ± 41.4**	1722.8 ± 47.8**
PAI-1	8591.6 ± 397	9104.5 ± 127	12302.5 ± 509**	10149.6 ± 221**	12323.1 ± 838**
Glucagon	66.8 ± 0.7	66.1 ± 0.9	122.7 ± 2.9**	68.3 ± 1.4	71.0 ± 1.3**
Insulin	94.7 ± 3.5	59.9 ± 3.4**	160.6 ± 5.7**	144.9 ± 15.5**	212.3 ± 9.7**
Pep-C	10217.9 ± 176	8472.2 ± 228**	11728.1 ± 289**	12656.6 ± 312**	16711.3 ± 284**
GIP	61.2 ± 2.4	45.9 ± 0.5**	59.1 ± 1.6	43.1 ± 1.1**	94.0 ± 4.1**
GLP-1	93.0 ± 1.9	92.4 ± 3.9	139.5 ± 2.7**	98.9 ± 5.2	92.0 ± 2.3
DPPIV	34.9 ± 1.4	26.9 ± 1.7*	29.9 ± 0.3*	33.8 ± 1.3	30.68 ± 1.18

^a^BMI = Body mass index. NM = normal weight(BMI = 18.5 to 24.9 kg/m^2^); OW = overweight(BMI ≥ 25.0 kg/m^2^); ObI = obesity(BMI = 30.o to 34.9 kg/m^2^); ObII/III(BMI = 35 to ≥ 40.0 kg/m^2^). PAI-1 = Plasminogen activator inhibitor; Pep-C = peptide-C; GIP = glucose dependent insulinotropic polypeptide; GLP-1 = Glucagon-like peptide-1; DPPIV = Dipeptidyl peptidase IV. Values represent the mean of triplicate ± error deviations(ED). Statistical differences were determined by Bonferroni test at *p* ≤ 0.01(*) and at *p* ≤ 0.001(**).

**Table 2:** Differentially accumulated proteins not reported in the HDDP lists that were identified in Mexican patients with diabetes.

ID	Spots in DIGE gels^a^	Name of Protein^b^	Accession Number^c^	Tendency^d^
**Low-abundance protein fraction**				
1	3, 8, 10, 29, 42, 57	Alpha-1B-glycoprotein	A1BG_HUMAN	Di Dd
2	56	Alpha-2-antiplasmin	A2AP_HUMAN	Dd
3	25, 34, 41	Leucine-rich alpha-2-glycoprotein	A2GL_HUMAN	Di Dd
4	5U, 34, 9, 25, 13, 5729, 47,	Alpha-1-antichymotrypsin	AACT_HUMAN	Oi, Di Dd, P
5	53	AMBP	AMBP_HUMAN	P
6	29, 57	Antithrombin-III	ANT3_HUMAN	Dd
7	40, 47, 51	Beta-2-glycoprotein 1	APOH_HUMAN	Di
8	1, 9, 4, 1115, 39, 55	Coiled-coil domain-containing protein 87	CCD87_HUMAN	Di P DOi Od Oi
9	1431, 35	Clusterin	CLUS_HUMAN	P Di
10	3, 36, 5341	Complement C4-A	CO4A_HUMAN	Di Dd
11	8, 22, 2845	Complement C4-B	CO4B_HUMAN	Di DOd
12	3637	Glutathione peroxidase 3	GPX3_HUMAN	Di Dd
13	3, 10, 25, 29, 45, 13, 36, 44, 15, 16, 22, 28, 41, 46U, 47, 48, 57	Hemopexin	HEMO_HUMAN	Di DOd, Od Oi, P, DOi, Dd
14	31	Histidine-rich glycoprotein	HRG_HUMAN	Di
15	48	Plasma protease C1 inhibitor	IC1_HUMAN	DOi
16	11, 32, 52, 36, 42, 5653	Inter-alpha-trypsin inhibitor heavy chain H4	ITIH4_HUMAN	DOi Di Dd P
17	9, 14, 29, 47, 32, 42, 5752	Kininogen-1	KNG1_HUMAN	Di P DOi Dd DOi
18	48	Lymphoid-restricted membrane protein	LRMP_HUMAN	DOi
19	36	MAGUK p55 subfamily member 2	MPP2_HUMAN	Di
20	10	Rhox homeobox family member 1	RHXF1_HUMAN	Di
**High-Abundance protein fraction**				
21	26U, 37 36	Zinc-alpha-2-glycoprotein	ZA2G_HUMAN	Dd Di
22	22 24	14-3-3 protein beta/alpha	1433B_HUMAN	Di Dd
23	14	Arf-GAP with SH3 domain, ANK repeat and PH domain-containing protein 2	ASAP2_HUMAN	Di
24	34	Haptoglobin-related protein	HPTR_HUMAN	Dd
25	8	Oncoprotein-induced transcript 3 protein	OIT3_HUMAN	Di
26	37	Otopetrin-3	OTOP3_HUMAN	Od
27	43	Trinucleotide repeat-containing gene 6C protein	TNR6C_HUMAN	Dd

^a^Spots numbers as indicated in Supplementary Files 1–3 where details including protein score, ion score, and peptide coverage are provided.

^b^Name of the protein and ^c^Accession number according to *Homo sapiens*(human) database from SwissProt(2015).

^d^Tendency of protein accumulation in different diabetic groups as indicated in Supplementary Files 1–3.

D = diabetes; O = obesity; DO = diabetes and obesity; P = punctual variation; i = increases; d = decreases.
