# Supplementary material for: 2D-DIGE as a strategy to identify serum biomarkers in Mexican patients with Type-2 diabetes with different body mass index
Source: Sci Rep. 2017 Apr 20;7:46536. doi: 10.1038/srep46536 (PMC5397846; doi:10.1038/srep46536)
Supplement: Supplementary Information [file srep46536-s1.doc]

**2D-DIGE as a strategy to identify serum biomarkers in Mexican patients with Type-2 diabetes with different body mass index**

Erik E. Gómez-Cardona, Eric E. Hernández-Domínguez, Aída J. Velarde-Salcedo, Alberto-Barrera-Pacheco, Agustín Diaz-Gois, Antonio De León-Rodríguez, & Ana P. Barba de la Rosa

**Supplementary Information**

**Supplementary Table S1.** Physical characteristics of Mexican patients with diabetes and control group

| **Group** | **n** | **Age**  **(years)** | **Female/Male proportion** | **Total protein^a^**  **(mg/mL)** |
| --- | --- | --- | --- | --- |
| **Control** | 23 | 39.1 ±10.6 | 10/13 | 73.8 ±0.9 |
| **Patients with diabetes** |  |  |  |  |
| Normal weight | 15 | 62.6 ±7.7 | 10/5 | 67.4 ±4.9 |
| Overweight | 22 | 56.0 ±5.9 | 12/10 | 73.7 ±3.3 |
| Obesity I | 13 | 57.1 ±7.9 | 7/6 | 66.6 ±0.4 |
| Obesity II/III | 12 | 59.5 ±7.6 | 11/2 | 64.6 ±2.0 |

^a^Total protein in crude serum

**Supplementary Table S2**. Sample combinations employed for 2D-DIGE gels

| **Gel** | **Cy2** | **Cy3** | **Cy5** |
| --- | --- | --- | --- |
| Gel 1 | Internal Standard | Normal weight | Control |
| Gel 2 | Internal Standard | Control | Obesity II/III |
| Gel 3 | Internal Standard | Obesity I | Overweight |
| Gel 4 | Internal Standard | Control | Obesity I |
| Gel 5 | Internal Standard | Overweight | Control |
| Gel 6 | Internal Standard | Obesity II/III | Normal weight |

**Supplementary Figure S1.** Comparison of the electrophoretic profiles of serum samples before and after depletion of the 14 most abundance proteins in serum. A) 1-DE profile in SDS-PAGE, Line 1=molecular weight marker, Lane 2=complete serum, Lane 3=abundance proteins fraction; Lane=4 non-abundance proteins fraction. B) 2-DE profiles of complete serum, C) 2-DE profile of abundance proteins fraction, and D) 2-DE profile of non-abundance protein fractions. For complete serum 1 mg of protein and Coomassie Brilliant Blue staining were employed and for the fractions 50 μg of protein and DIGE labelling were used.

**Supplementary Figure S2.** Classification of all differentially accumulated proteins identified in both fractions. The charts present the distribution of the proteins identified in this study based on its A) Molecular Function and B) Biological Process in which they are involved according to Gene Ontology ([http://www.geneontology.org](http://www.geneontology.org/)).

**Supplementary Figure S3.** Tendencies observed for the differential spots and its association to the variants on the study.

This tendencies were evaluated on the clusters generated by the pattern/partition analysis. Graphics were obtained on the

Differential expression analysis section as part of the Extended data analysis.

**Supplementary Figure S4.** Expression level of 14-3-3 protein at individual level. A) Serum levels of 14-3-3 protein examined by WB in individual serum samples. B) Densitometry analysis normalized with total protein. The graph shows the average log-standardized abundance of the bands detected by WB against 14-3-3 protein for each patient in the groups. The bars indicate standard error. Asterisks show significant differences at *p*<0.001. Full-length blots are presented in Supplementary Figure S5.

**Supplementary Figure S5.** Full-length western blots for biomarker validation step. A) Uncropped images included in candidate biomarker validation of the groups showed in Figure 4. B) Complete image of the individual western blot for 14-3-3 showed in Supplementary Figure S4. Std=Pooled internal standard for sample normalization; Ctrl=Control; NW=Normoweigth; OW= Overweigth; Ob I= Obesity I; ObII/III=Obesity II/III.
